# CSF3 Is a Potential Drug Target for the Treatment of COVID-19

**DOI:** 10.3389/fphys.2020.605792

**Published:** 2021-01-22

**Authors:** Chao Fang, Jie Mei, Huixiang Tian, Yu-Ligh Liou, Dingchao Rong, Wei Zhang, Qianjin Liao, Nayiyuan Wu

**Affiliations:** ^1^Hunan Key Laboratory of Pharmacogenetics, Department of Clinical Pharmacology, Xiangya Hospital, Institute of Clinical Pharmacology, Central South University, Changsha, China; ^2^Hunan Cancer Hospital, The Affiliated Cancer Hospital of Xiangya School of Medicine, Central South University, Changsha, China; ^3^Department of Pharmacy, Xiangya Hospital, Central South University, Changsha, China; ^4^Department of Spine Surgery, Xiangya Hospital, Central South University, Changsha, China

**Keywords:** COVID-19, MOE, ritonavir, elbasvir, CSF3

## Abstract

Coronavirus Disease 2019 (COVID-19) is an acute respiratory infectious disease that appeared at the end of 2019. As of July 2020, the cumulative number of infections and deaths have exceeded 15 million and 630,000, respectively. And new cases are increasing. There are still many difficulties surrounding research on the mechanism and development of therapeutic vaccines. It is urgent to explore the pathogenic mechanism of viruses to help prevent and treat COVID-19. In our study, we downloaded two datasets related to COVID-19 (GSE150819 and GSE147507). By analyzing the high-throughput expression matrix of uninfected human bronchial organoids and infected human bronchial organoids in the GSE150819, 456 differentially expressed genes (DEGs) were identified, which were mainly enriched in the cytokine–cytokine receptor interaction pathway and so on. We also constructed the protein–protein interaction (PPI) network of DEGs to identify the hub genes. Then we analyzed GSE147507, which contained lung adenocarcinoma cell lines (A549 and Calu3) and the primary bronchial epithelial cell line (NHBE), obtaining 799, 460, and 46 DEGs, respectively. The results showed that in human bronchial organoids, A549, Calu3, and NHBE samples infected with SARS-CoV-2, only one upregulated gene CSF3 was identified. Interestingly, CSF3 is one of the hub genes we previously screened in GSE150819, suggesting that CSF3 may be a potential drug target. Further, we screened potential drugs targeting CSF3 by MOE; the top 50 drugs were screened by flexible docking and rigid docking, with 37 intersections. Two antiviral drugs (Elbasvir and Ritonavir) were included; Elbasvir and Ritonavir formed van der Waals (VDW) interactions with surrounding residues to bind with CSF3, and Elbasvir and Ritonavir significantly inhibited CSF3 protein expression.

## Introduction

Coronavirus Disease 2019, first detected in Wuhan, China, in December 2019, has been raging around the world from its detection through to current times (Kannan et al., 2020; Thompson, 2020). On January 9, 2020, the World Health Organization (WHO) announced the discovery of the new coronavirus. This coronavirus was originally called 2019-nCoV and then officially named SARS-CoV-2, which had never been found among humans. On February 11, 2020, the respiratory disease originating from SARS-CoV-2 infection was called COVID-19 (Lu et al., 2020; Mahase, 2020). Although the virus was first discovered in Wuhan, there is still controversy about its specific origin (Andersen et al., 2020; Forster et al., 2020). WHO announced that COVID-19 had become a global health problem because it has spread in more than 140 countries around the world (Zhai et al., 2020).

Fever, sore throat, fatigue, cough, or dyspnea are the typical symptoms of COVID-19 (Kim et al., 2020; Ozma et al., 2020). As of 10 August 2020, confirmed COVID-19 cases have reached 19,718,030, including 728,013 deaths in the world^1^. The government shared the genome sequence of SARS-CoV-2 with the public in a timely manner. This was effective in helping scientists deal with the emergency (Harcourt et al., 2020; Wu et al., 2020). Although most countries in the world have effectively controlled COVID-19, it is still far from being completely over. This is because research on the treatment and prevention of COVID-19 is still insufficient, and the vaccine under research has not yet been fully applied in clinic (Dhama et al., 2020).

At present, the treatment of COVID-19 is still limited, and it has been reported that some drugs may have a therapeutic effect on COVID-19. As an antimalarial agent, chloroquine and its derivative, hydroxychloroquine, have shown to be active against COVID-19 *in vitro* (Wang M. et al., 2020). However, there is not enough clinical data to support the therapeutic effect of chloroquine or hydroxychloroquine on COVID-19 (Chen et al., 2020; Huang et al., 2020).

In addition, remdesivi (Jean et al., 2020), lopinavir, and emetine (Choy et al., 2020) have been reported to inhibit SARS-CoV-2 *in vitro*. But these drugs are not currently recommended for clinical use, and are only recommended for use in clinical trials (Kotwani and Gandra, 2020). Moreover, research on the screening of anti-SARS-CoV-2 drugs and their mechanism is mainly focused on angiotensin-converting enzyme 2 (ACE2) (Williamson and Kerimi, 2020). Whether there are other drug targets for SARS-CoV-2 remains an unanswered question for researchers.

In our study, we analyzed the high-throughput sequencing data of uninfected human bronchial organoids and SARS-CoV2 infected human bronchial organoids in the GEO database to explore the effects of SARS-CoV2 (Blanco-Melo et al., 2020). Then we verified the high-throughput data of A549, Calu3, and NHBE cell lines, finding that only CSF3 (Tamada et al., 2006) was up-regulated in the four sets of samples. It has been reported that the granulocyte colony-stimulating factor (CSF3) was the most upregulated gene after SARS-CoV2-infected genes (Nunnari et al., 2020), suggesting that CSF3 is a significant target after infection and it may also be a potential target for drug therapy. Therefore, we have screened drugs that may potentially target CSF3 for the treatment of COVID-19 from the FDA-approved drug library.

## Materials and Methods

### Data Download

The Gene Expression Omnibus (GEO)^2^ database was used to downloaded datasets about COVID-19. With “COVID-19 OR SARS-CoV-2, Homo sapiens” as the screening criteria, we selected GSE150819 and GSE147507 for analysis. In GSE150819, human bronchial organoids (hBO) from commercially available cryopreserved primary human bronchial epithelial cells (hBEpC) were constructed for severe acute respiratory syndrome coronavirus 2 (SARS-CoV-2) research. We chose three uninfected samples and three SARS-CoV-2 infected samples to analyze. In GSE147507, we selected primary human lung epithelium cells (NHBE) and lung adenocarcinoma cells (A549, Calu-3), which were mock treated or infected with SARS-CoV-2.

### Identification of DEGs

In our study, | log fold change (FC)| > 1.5 and *p* < 0.05 were used as the cut-off criteria to find DEGs in uninfected and SARS-CoV-2-infected organoids or cell lines. Then, these DEGs were visualized by volcano plots and heatmaps, which were performed by R package in RStudio. VENNY 2.1.0^3^ online software was used to identify overlapping DEGs in the four sets of samples.

### GO and KEGG Enrichment Analysis

Gene Ontology (GO) analysis classified the genes in the difference tables according to their functions, which included biological process (BP), cellular component (CC), and molecular function (MF). The Kyoto Encyclopedia of Genes and Genomes (KEGG) pathway enrichment analysis showed the key pathways of clustered genes. GO and KEGG enrichment analysis of DEGs in GSE150819 were performed by the Database for Annotation, Visualization, and Integrated Discovery (Huang et al., 2009) (DAVID)^4^ v6.8. And a *p* < 0.05 was considered to be statistically significant.

### Protein–Protein Interaction (PPI) Network Construction, Module Analysis, and Identification of Hub Genes

In order to facilitate understanding of the interaction between the proteins corresponding to these DEGs, a PPI network was constructed by Search Tool for the Retrieval of Interacting Gene (STRING)^5^ database (Szklarczyk et al., 2015). In the network, core modules and top 10 hub genes were identified by ClusterONE and CytoHubba in Cytoscape software, suggesting the key genes and modules which might play a significant role in COVID-19.

### Gene Set Enrichment Analysis

Gene set enrichment analysis (GSEA) is a software which can analyze the positive and negative regulatory pathway of a hub gene or all DEGs. To better understand the role of CSF3 in COVID-19, we chose KEGG function of CSF3 in GSE10819 to analyze, obtaining the key positive and negative regulatory pathways by NOM *p*-value ranking (*p* < 0.05).

### Molecular Docking

The X-ray structure of CSF3 was downloaded from RCSB Protein Data Bank (PDB ID: 2D9Q)^6^. The FDA-approved drug library was obtained from DrugBank^7^. The 2D structures of the FDA-approved drug molecules were drawn in ChemBioDraw 2014 and converted to 3D in MOE through energy minimization. MOE dock was used for homology modeling and molecular docking simulations of the binding affinity between FDA-approved drugs and the target protein. Prior to docking, the force field of AMBER10:EHT and the implicit solvation model of Reaction Field (R-field) were selected. The protonation state of the protein and the orientation of the hydrogens were optimized by LigX, at a PH of 7 and temperature of 300 K. The docking workflow followed the “induced fit” protocol, in which the side chains of the receptor pocket were allowed to move according to ligand conformations, with a constraint on their positions. The weight used for tethering side chain atoms to their original positions was 10. All docked poses were ranked by London dG scoring first, then a force field refinement was carried out on the top 10 poses followed by a rescoring of GBVI/WSA dG. The conformation with the lowest free energy of binding was selected as the best (probable) binding mode. Molecular graphics were generated by PyMOL.

### Western Blotting

Crude cellular proteins were separated by SDS-PAGE and transferred to nitrocellulose membranes. Membranes were blocked in 5% non-fat milk and probed with primary antibodies (1:1,000 dilution) against CSF3 (Abcam, ab181053) overnight at 4°C. Membranes were incubated with horseradish peroxidase (HRP)-conjugated secondary antibodies for 1.5 h and immunolabeling detected using a Bio-Rad imaging system.

## Results

### Identification of DEGs in Datasets

**FIGURE 1:**
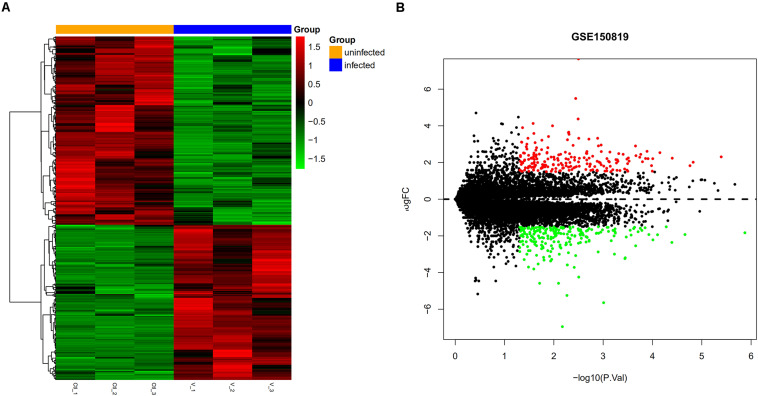
Visual expression of DEGs in GSE150819. **(A)** The heatmap of DEGSs. **(B)** The volcano plot of DEGs. Red and green represent relative upregulation and downregulation of gene expression, respectively.

In GSE150819, we analyzed three samples of uninfected human bronchial organoids and three samples of SARS-CoV-2-infected human bronchial organoids, obtaining 456 DEGs, of which 205 were upregulated DEGs and 251 downregulated DEGs (Table 1). To visualize the expression of the DEGs in GSE150819, a heatmap and volcano plot were constructed, as shown in Figure 1.

**TABLE 1:** The consensual DEGs were identified in GSE150819.

Groups	Gene symbol
Up-regulated	ALG1L2, ICAM1, TAF7L, SPRR2B, TSPAN18, LGALS2, TRABD2A, VNN1, KLK6, SLC7A7, POU2F2, NT5E, ESM1, POPDC3, NOXO1, IL7R, SLC5A1, HLA-F, C10orf10, SSC4D, SELL, LINC00514, LRRC25, PTPN20, SNORA41, STC2, ALDOB, PLAU, SAA2, ZNF114, IL36G, ART3, SPRR2D, PARVB, MUC13, LIF, NES, ECSCR, FAM205A, ADRA1B, SH2D2A, TNFRSF9, RASD1, EPSTI1, AWAT2, ADGRL4, SAA4, XDH, MMP7, RND1, IL4I1, OOEP, RAB42, TNIP3, ANXA2P3, NOX1, ADAMTS6, PSG9, UBD, WNT7A, MPZ, GALNT13, KIRREL3, SNORD24, IL32, MT2A, BCL2A1, TRPV2, NTNG1, SV2B, LOC102723649, FCGR1A, MYCT1, SYNGR3, DIO3, IDO2, IDO1, SNAI1, OLFM1, RNASE7, C15orf48, KLK5, HEPHL1, KCNN3, SAA1, HMGN2P46, APOBEC3A, SYNDIG1, PRNCR1, ROBO4, SLC5A8, IFITM1, B4GALNT2, CUX2, COL22A1, GDF15, L1CAM, LINC00880, CXCL8, IL19, TFF1, MSC-AS1, GFPT2, PLA2G4C, CXCL2, IFIT3, CHAC1, TRHDE, ATRNL1, G0S2, GBP5, C8orf4, MYH16, PYDC1, SYNPR-AS1, ZNF385C, SLC7A2, MMP9, IL17C, MCHR1, IL1A, CEACAM7, PDE4B, IL12RB1, SMOC1, COL13A1, PITX3, MRGPRX3, CCL20, C19orf38, SULT1C2, SERPINA3, ALPL, NCF1B, EPS8L3, IFI27, SPRR2A, CD69, BEAN1, SP140, DLGAP3, IL10, LINC00323, LOC101929626, MIR765, PCBP3, THSD7B, AMZ1, PDGFB, SPRR2E, DGCR9, BATF3, SLC5A5, HERC5, DUOXA2, EBI3, CYP27A1, SLC26A4-AS1, SERPINE1, CXCL3, INHBA, LRRC55, MX1, LOC101929427, C4orf26, ISG15, CDH19, DSCAM, CD177, MSC, SLCO4A1, KISS1, C1QTNF1, IL17REL, RAB33A, IFI6, AKR1D1, ASB4, CCIN, PLCG1-AS1, LEFTY1, CXCL10, SERPINA5, CXCL5, SPRR2F, FFAR2, KCNK3, GAL, SERPINA9, GCGR, CCL5, IL1B, GPHA2, PLA2G7, UGT1A4, IL23A, SLC26A4, KIRREL2, CXCL11, DEFB4A, RSAD2, TNFRSF1B, ZBP1, CSF3, FDCSP, PRSS1
Down-regulated	KRT1, DIO2, DSG1, KRT3, LOC101929574, NPTX1, SNORD116-22, GRIK3, BSND, CASP1P2, SLC16A6, LUZP2, CYP4F22, AACSP1, COL14A1, PDZRN4, SPINK7, ANGPTL2, KCNJ16, ITPR1-AS1, TDO2, PCDHB19P, COL3A1, LOC101927311, LOC100652999, RNU11, RRH, SNORD9, TMCO5A, TRIM34, KRT4, LOC100506834, SRD5A2, CADM2, KCNH2, ABCD2, CACNA1E, FAM13C, FLJ43879, LOC100505795, LOC101927354, C1QTNF8, CRNN, LOC101928118, AQP6, HMGCS2, FGF22, HYPK, INTS4P2, PCDHA5, RADIL, SNORD16, VIPR1-AS1, GLI1, SLC6A4, SPARCL1, LOC100505915, KRTDAP, LINC01583, FRMPD3, PTPRT, LOC653653, TLX1NB, HIST1H1D, PTPRQ, ADH1A, NHSL2, TSLP, ELN, ADGRG3, ATP13A4-AS1, BASP1P1, BPIFC, CATSPERD, CCL27, COL18A1-AS1, DRD2, GABRQ, GDF6, LINC00111, LOC100132781, LOC101927954, MIR34C, PGLYRP2, RNU6-79P, RTP1, SNORD77, DIAPH2-AS1, PIANP, WASH5P, CEMIP, NBPF11, POU3F1, TNNT2, CCDC152, NGB, CRISP3, EYS, BCHE, BEND6, LOC101927881, LOC284825, RIMBP2, PLXDC1, EPB41L3, RPTN, HMCN1, CCDC36, SNORD59B, APCDD1, SDR9C7, C8orf34, AJAP1, LOC101928505, MEIS1-AS2, PPP1R42, MAP7D2, IGFBP5, FAT3, PSAPL1, PRKCB, STEAP3-AS1, EVPLL, KRT15, ANPEP, BGN, DPH6-AS1, RGL2, RGPD3, POSTN, SLURP1, SLC10A5, EPHA3, ASXL3, MPPED2, SHISA9, C1orf168, SLC1A3, RBM20, SNHG25, TMEM211, MANEA-AS1, WDR17, KRT13, AADAC, CCDC136, PPP1R16B, SBSN, RPL3L, TNFAIP8L2, VASH2, RERG, ANXA10, CACNA1D, IGFL1, KRT10, DLGAP1, LOC101928865, ONECUT3, DCLK1, CCDC73, ELFN1, FAM66A, HYDIN2, JAM2, LINC00871, LOC101929441, MIR6739, AR, HTR2B, SLC24A3, LOC730101, GLI2, GRM4, HLF, TNNI2, SYNPO2, GPR20, KANTR, IGFL2, MIR573, SNORA77, MESP1, LOC284009, PCDH18, CLDN8, FAM46C, DAPL1, LINC01121, UPK1A, CXCL14, GPC3, SLC47A2, LAX1, MT1G, SCML2, CCDC3, TPTE2P1, COL21A1, RTCA-AS1, SOAT2, CNR1, LY6H, ZNF157, GUSBP9, MIR4523, DMRT3, GSTA2, EPGN, P2RY1, RDH12, LINC01116, ANKH, C14orf132, KY, LINC00624, METTL21EP, MIR320E, MIR4653, SCGB2B2, SMAD5-AS1, SMIM2-AS1, TAS2R14, APOBEC2, SAMD5, TMPRSS11B, FOXP2, SPOCK2, TP73, SOSTDC1, H19, CYP2C8, BEND5, ARHGEF33, FAM25A, NKAPP1, OR7E47P, TMEM220-AS1, IL2RB, CLIC5, SNX31, TMEM200A, PAK7, PDE6A, DNAJC22, CILP, LOC200772, TMEM56-RWDD3, COLCA1, KRT6C

### Enrichment Analysis of DEGs

To identify the significant pathways associated with the DEGs between uninfected human bronchial organoids and infected human bronchial organoids in GSE150819, we performed the GO and KEGG pathway enrichment analysis by DAVID.

The result showed that in GO enrichment analysis (Table 2), genes were enriched in biological processes (BP) of inflammatory response, immune response, and cell chemotaxis. As for cellular component (CC), these genes showed enrichment in extracellular space, extracellular region, and integral component of plasma membrane. The molecular function (MF) results showed cytokine activity, chemokine activity, and structural molecule activity. KEGG pathway enrichment analysis was shown in Table 2. DEGs were mainly enriched in cytokine receptor interaction, TNF signaling pathway, and African trypanosomiasis.

**TABLE 2:** GO and KEGG enrichment analysis of DEGs.

Category	ID	Term	Count	*P*-values
**GO functional enrichment analysis of DEGs**				
BP	GO:0006954	Inflammatory response	24	1.28E–06
BP	GO:0006955	Immune response	25	2.25E–06
BP	GO:0060326	Cell chemotaxis	10	4.00E–06
BP	GO:0006935	Chemotaxis	13	4.10E–06
BP	GO:0007267	Cell–cell signaling	17	3.47E–05
CC	GO:0005615	Extracellular space	69	1.25E–13
CC	GO:0005576	Extracellular region	76	3.18E–13
CC	GO:0005887	Integral component of plasma membrane	57	1.66E–07
CC	GO:0005578	Proteinaceous extracellular matrix	18	1.80E–05
CC	GO:0001533	Cornified envelope	7	2.36E–04
MF	GO:0005125	Cytokine activity	18	4.29E–08
MF	GO:0008009	Chemokine activity	10	2.98E–07
MF	GO:0005198	Structural molecule activity	17	2.06E–05
MF	GO:0004833	Tryptophan 2,3-dioxygenase activity	3	0.001061212
MF	GO:0008083	Growth factor activity	11	0.0010628
**KEGG pathway enrichment analysis of DEGs**				
KEGG_PATHWAY	hsa04060	Cytokine–cytokine receptor interaction	24	1.92E–10
KEGG_PATHWAY	hsa04668	TNF signaling pathway	10	2.20E–04
KEGG_PATHWAY	hsa05143	African trypanosomiasis	6	3.98E–04
KEGG_PATHWAY	hsa04974	Protein digestion and absorption	8	0.001555896
KEGG_PATHWAY	hsa05323	Rheumatoid arthritis	8	0.001555896

### Construction of PPI Network and Identification of Hub Genes

**FIGURE 2:**
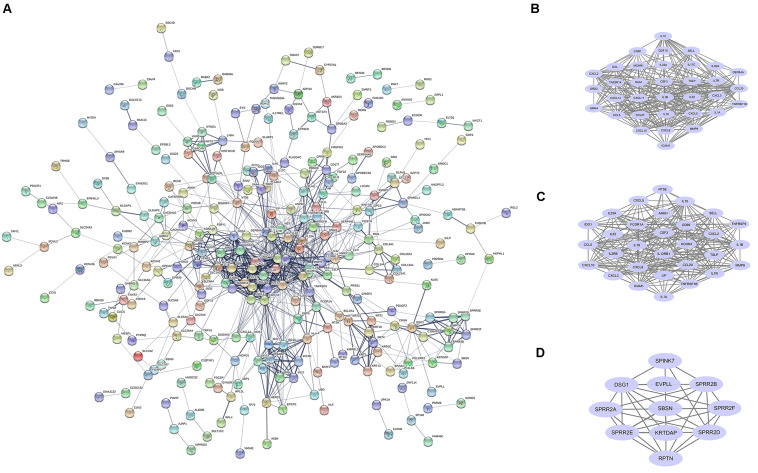
PPI network and core modules analysis of DEGs in GSE150819. **(A)** The PPI network of DEGs was constructed by STRING. **(B–D)** ClusterONE identified the core modules in the PPI network.

STRING online software constructed the PPI network of 456 DEGs, composed of 282 nodes and 930 edges after excluding the isolated nodes, as shown in Figure 2A. ClusterONE identified the top three modules in the PPI network, suggesting that these submodules might be highly significant in the process of COVID-19. Module 1 (Figure 2B) and module 2 (Figure 2C), consisting of 22 and 12 DEGs, respectively, were both enriched in KEGG pathway, including the Cytokine–cytokine receptor interaction and TNF signaling pathway. Module 3 (Figure 2D) contained 14 nodes and 45 edges.

The top 10 hub genes were also calculated by the Degree algorithm in CytoHubba. They were MMP9, CCL20, CSF3, CCL5, CXCL10, IL1B, SAA1, ICAM1, IL10, and CXCL8 (Figure 2D). Interestingly, all of them were upregulated after being infected with SARS-CoV-2.

### Identification of Overlapping Genes in Different Datasets and GSEA Analysis

**FIGURE 3:**
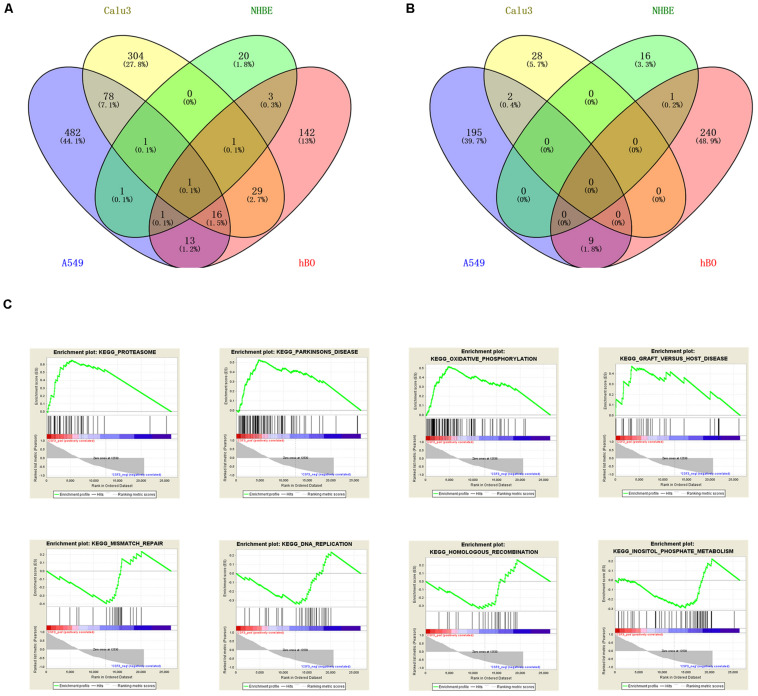
Identification and analysis of overlapping DEGs. VENNY software identified the upregulated DEGs **(A)** and downregulated DEGs **(B)** of the four sets of samples in GSE150819 and GSE147507. **(C)** The overlapping gene CSF3 was analyzed by GSEA. There were four positive regulatory pathways of CSF3, which were proteasome, Parkinson’s disease, oxidative phosphorylation, and Graft vs. host disease. The following were four negative regulatory pathways of CSF3: mismatch repair, DNA replication, homologous recombination, and inositol phosphate metabolism.

Similarly, we also analyzed the three cell lines samples, which were NHBE, A549, and Calu3, and their SARS-CoV-2-infected cells in GSE147507. After screening, the results suggested that there were 45 DEGs in the NHBE group, 158 DEGs in the A549 group, and 460 DEGs in the Calu3 group. VENNY 2.1 online software was used to find the overlapping DEGs in the four sets of samples, and finally only one overlapping DEG was found, as shown in Figures 3A,B. Only CSF3 showed a common upregulated gene in infected human bronchial organoids and cell lines.

For GSEA, we analyzed CSF3 enrichment in GSE150819 to explore its potential molecular functions, obtaining the positive and negative regulatory pathways of CSF3. The results were shown in Figure 3C. The results showed that the top four positive regulatory pathways of CSF3 were proteasome, Parkinson’s disease, oxidative phosphorylation, and Graft vs. host disease. While the top four negative regulatory pathways of CSF3 were mismatch repair, DNA replication, homologous recombination, and inositol phosphate metabolism.

### Using CSF3 as the Target to Find Potential Targeted Drugs

Giuseppe Nunnari discovered the potential value of CSF3 in COVID-19 research (Nunnari et al., 2020). We obtained the X-ray structure of CSF3 (Tamada et al., 2006) from the RCSB PDB and aimed to screen out the 2,470 FDA-approved drugs to potentially target CSF3. We investigated the binding mode of FDA-approved drugs with CSF3 by rigid and flexible docking simulation. The scores of the top 50 in flexible docking are shown in Table 3. Among them, ubiquinol (DB11340), an active antioxidant (Ernster and Forsmark-Andrée, 1993; Sies, 1997), got the highest score of -8.0513706 kcal/mol. In addition, two antiviral drugs, Elbasvir (DB11574) (Myers et al., 2015) with scores of -6.5620656 kcal/mol and Ritonavir (DB00503) (Cameron et al., 1998) with scores of -6.259393 kcal/mol, have attracted our attention.

**TABLE 3:** KEGG pathway analysis of DEGs.

Category	Term		Count	*P*-values
KEGG_PATHWAY	hsa04060	Cytokine–cytokine receptor interaction	24	1.92E–10
KEGG_PATHWAY	hsa04668	TNF signaling pathway	10	2.20E–04
KEGG_PATHWAY	hsa05143	African trypanosomiasis	6	3.98E–04
KEGG_PATHWAY	hsa04974	Protein digestion and absorption	8	0.001555896
KEGG_PATHWAY	hsa05323	Rheumatoid arthritis	8	0.001555896

**FIGURE 4:**
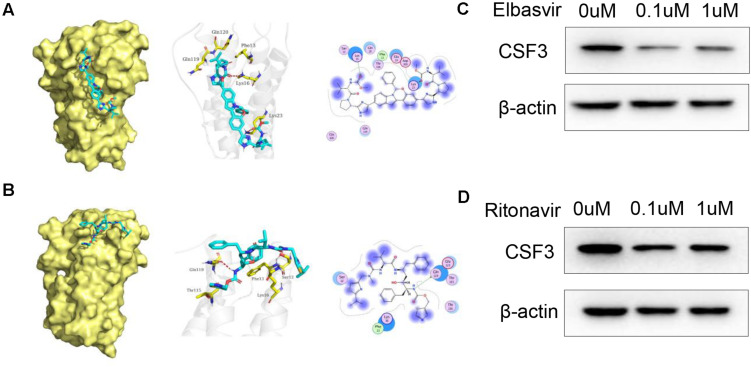
Docked pose of CSF3 with Elbasvir and Ritonavir. **(A)** Overall structural view of CSF3 with Elbasvir, wherein Elbasvir are chimeric into protein; Detailed interaction view between Elbasvir and CSF3 wherein hydrogen bonding is shown as a red dashed line; 2D interaction view between Elbasvir and CSF3, with green arrows representing hydrogen bonding interactions. **(B)** Overall structural view of CSF3 with Ritonavir, wherein Ritonavir are chimeric into protein; Detailed interaction view between Ritonavir and CSF3 wherein hydrogen bonding is shown as red dashed line; 2D interaction view between Ritonavir and CSF3, with green arrows representing hydrogen bonding interactions. **(C,D)** Both Elbasvir and Ritonavir inhibited CSF3 expression in A549 cells when treated with 0.1 and 1 μM of Elbasvir/Ritonavir for 24 h.

The binding mode of Elbasvir with CSF3 were illustrated in Figure 4A. Elbasvir has formed a suitable steric complementarity with the binding site of CSF3. The oxygen atom of carbonyl in Elbasvir, regarded as hydrogen bond acceptors, form the hydrogen bonds with Lys 16 and Lys 23 in CSF3. The binding mode of Ritonavir with CSF3 were illustrated in Figure 4B. The nitrogen atom in Ritonavir, regarded as a hydrogen bond donor, forms hydrogen bonds with Gln 119 in CSF3. Moreover, Elbasvir and Ritonavir formed van der Waals (VDW) interactions with surrounding residues, which mainly contribute to the binding energy between Elbasvir and Ritonavir with CSF3.

We further verified the effect of Elbasvir and Ritonavir on CSF3; WB results show that both Elbasvir and Ritonavir significantly inhibited the CSF3 protein expression in A549 cells (Figures 4C,D).

## Discussion

In the current global pandemic, COVID-19 has infected more than 19 million people and caused more than 720,000 deaths. It has a very high infection rate (Li R. et al., 2020), and the case fatality rate has been reported to be as high as 5% (Li L.Q. et al., 2020). Clinical data in the United States showed that among patients hospitalized in the intensive care unit, case fatality was up to 40% (Wiersinga et al., 2020). The virus has also bene shown to spread among people of all ages^8^. Studies have shown that the risk of death is highly correlated with the age gradient, and younger age may be associated with lower mortality (Verity et al., 2020).

This is an epidemic that has affected all mankind and requires our joint efforts. In the winter when the virus was raging (Smit et al., 2020), China quickly quarantined Wuhan, promptly and effectively suppressing the spread of COVID-19. The Chinese people came together with the government to combat the disease. As of March 2020^9^, the daily number of local cases in mainland China has dropped to two digits, and the infected are mainly from people returning from abroad.

But the virus still exists, and we still have not completely defeated it. The existing obstacles are the limitations of treatment methods, the lack of effective drugs (Triggle et al., 2020), the difficulty of vaccine development (Uddin et al., 2020), and the inability to completely block the spread of the virus (Rothan and Byrareddy, 2020). All of these issues require continuous efforts by researchers, and research into treatment and prevention should be given equal attention. On the one hand, reliable treatments and therapeutic drugs need to be found. On the other hand, the spread of the virus needs to be blocked and vaccines need to be researched as quickly as possible.

In our study, we hope to find potential drug therapeutic targets from the high-throughput data of virus-infected organoids and cells and explore whether there are clinical drugs in use that can target the infectious protein. We found 456 DEGs in the analysis of uninfected and SARS-CoV-2-infected human bronchial organoids. KEGG enrichment analysis showed that these genes were mainly enriched in Cytokine–cytokine receptor interaction and TNF signaling pathway. Through the verification of high-throughput data of A549, Calu3, and NHBE cell lines, we have found an important target protein CSF3, which, along with its receptor CSF3R, participate in the regulation of granulopoiesis, neutrophil function, and hematopoietic stem cell mobilization (Zhang et al., 2018). It has been reported that CSF3 mutations have carcinogenic effects (Hollmén et al., 2016; Jing et al., 2016). Virus-related research has found that human respiratory syncytial virus (hRSV) infection disrupted the polarity of the pediatric respiratory epithelial secretome and was associated with immune modulating proteins (CXCL6, CXCL16, and CSF3) never before linked with this virus before (Touzelet et al., 2020). The latest reports showed that CSF3 is the most modulated gene in NHBE cells infected with SARS-CoV-2 (Nunnari et al., 2020), indicating the potential value of CSF3.

Regarding CSF3 as a drug target, we use molecular docking simulation technology by MOE to screen out potential drugs from the FDA-approved drug library, which were downloaded from DrugBank. These drugs were scored separately by rigid docking and flexible docking. Among the top 50 drugs, a total of 37 drugs were screened by both methods. The screened drugs included anti-HCV drugs, such as Ombitasvir (DB09296) (Ascione et al., 2018), Daclatasvir (DB09102) (Lee, 2013), and Elbasvir (DB11574) (Myers et al., 2015), and the anti-HIV drugs, Cobicistat (DB09065) (Angione et al., 2018) and Ritonavir (DB00503) (Cameron et al., 1998). The drug with the highest score is Ubiquinol (DB11340), which is an active antioxidant drug (Ernster and Forsmark-Andrée, 1993). No antiviral drugs have been proven to be effective against COVID-19. Therefore, we give priority to antiviral drugs such as Elbasvir to explore whether it has a potential effect to COVID-19 in our study. Similarly, Junmei Wang screened out Elbasvir, carfilzomib, eravacycline, valrubicin, and lopinavir as potential inhibitors of SARS-CoV-2’s main protease (Wang, 2020). In Meenakshisundaram Balasubramaniam’s study, Elbasvir was predicted to bind multiple SARS-CoV-2 proteins, blocking virus replication, and had a good antiviral effect (Balasubramaniam and Reis, 2020). As an FDA-approved drug for anti-HIV and HCV, Ritonavir (Feld et al., 2014) was tried early in the treatment of COVID-19 (Lim et al., 2020; Wang Y. et al., 2020), although the results were not satisfactory (Griffin, 2020). At present, these predicted antiviral drugs are still not recommended for clinical use, and are only supported for clinical trials. In a word, further mechanism exploration and clinical trials are needed for the application of these antiviral drugs.

## Conclusion

In conclusion, our study identified the DEGs and underlying pathways in uninfected and SARS-CoV-2-infected human bronchial organoids and cell lines, found the only upregulated gene was CSF3, and explored its potential value. We regarded CSF3 as a potential drug target, and used molecular docking and virtual drug screening technology to find drugs that may interact with it to find potential treatments for COVID-19. Elbasvir, Ritonavir, and other antiviral drugs were screened through both rigid docking and flexible docking, and they were potential anti-COVID-19 drugs that have been studied. Targeting CSF3 may be a potential therapeutic mechanism of these drugs, but a more in-depth exploration is needed.

## Data Availability Statement

The original contributions presented in the study are included in the article/supplementary material, further inquiries can be directed to the corresponding author/s.

## Author Contributions

CF, NW, WZ, and Y-LL: conception and design. NW and QL: administrative support. JM, CF, and HT: collection and assembly of data. CF, DR, and JM: data analysis and interpretation. NW, JM, and CF: manuscript writing. All authors finally approved the manuscript.

## Conflict of Interest

The authors declare that the research was conducted in the absence of any commercial or financial relationships that could be construed as a potential conflict of interest.

## Footnotes

1 https://www.who.int/emergencies/diseases/novel-coronavirus-2019

2 https://www.ncbi.nlm.nih.gov/geo/

3 https://bioinfogp.cnb.csic.es/tools/venny/

4 https://david.ncifcrf.gov/

5 https://string-db.org/cgi/input.pl

6 http://www.rcsb.org/

7 https://www.drugbank.ca/

8 https://www.who.int/publications-detail/report-of-the-who-china-joint-mission-on-coronavirus-disease-2019-(covid-19)

9 https://www.who.int/docs/default-source/coronaviruse/situation-reports/20200331-sitrep-71-covid-19.pdf?sfvrsn=4360e92b_8
